# IF I COULD TURN BACK TIME: A THREE-YEAR LONGITUDINAL ASSESSMENT OF MUSIC ENJOYMENT IN OLDER ADULT HEARING AID USERS

**DOI:** 10.1093/geroni/igae098.2531

**Published:** 2024-12-31

**Authors:** Isaac Alter, Alexander Chern, Michael Denham, Alexis Leiderman, Jessica Galatioto, Jennifer Jones, Anil Lalwani

**Affiliations:** Columbia University, New York, New York, United States; Johns Hopkins University, Baltimore, Maryland, United States; Columbia University, New York, New York, United States; Elmhurst Hospital Center/Mount Sinai Hospital, Queens, New York, United States; Columbia University Irving Medical Center, New York, New York, United States; Columbia University Irving Medical Center, New York, New York, United States; Columbia University, New York, New York, United States

## Abstract

**Introduction:**

Hearing loss (HL) is remarkably prevalent among older individuals, and is associated with worsened music enjoyment and perception. We sought to examine longitudinal changes in music enjoyment among long-term hearing aid (HA) users.

**Methods:**

Adult HA users were recruited from a tertiary academic medical center and community setting. Participants reported their music listening habits and subjective music enjoyment using 10-point Likert scales, and underwent a melody discernment listening task, once between July-November 2020 and again three years later. Paired t-tests were used to compare participants’ responses between time points, and multiple linear regression was employed to investigate relationships between hearing levels (measured by pure tone average (PTA) of the better hearing ear) and music enjoyment.

**Results:**

Thirty-nine HA users participated, with an average (SD) age of 72.9 (7.8) and 24.3 (18.3) years of HA use. Despite a significant increase in PTA from 2020 to 2023 (47.2 to 52.6, p< 0.001), there was no significant change in time spent listening to music, overall music enjoyment, or melody discernment ability across the two time points. However, there was a significant association between change in PTA and music enjoyment, with a one-point worsening in PTA between 2020-2023 correlating to a 0.14-point decrease in music enjoyment (p=0.041).

**Conclusion:**

Our results suggest that despite worsening hearing over time, older HA users do not uniformly report reduction in music enjoyment or time spent listening to music, nor do their melody discrimination abilities diminish. However, more severe worsening in hearing may predict lower self-reported music enjoyment.

